# Load-Bearing Detection with Insole-Force Sensors Provides New Treatment Insights in Fragility Fractures of the Pelvis

**DOI:** 10.3390/jcm9082551

**Published:** 2020-08-06

**Authors:** Daniel Pfeufer, Christopher A. Becker, Leon Faust, Alexander M. Keppler, Marissa Stagg, Christian Kammerlander, Wolfgang Böcker, Carl Neuerburg

**Affiliations:** Department of General, Trauma and Reconstructive Surgery, University Hospital, Ludwig-Maximilians-University Munich, 81377 Munich, Germany; daniel.pfeufer@med.uni-muenchen.de (D.P.); leon.faust@med.uni-muenchen.de (L.F.); alexander.keppler@med.uni-muenchen.de (A.M.K.); marissa.stagg@med.uni-muenchen.de (M.S.); christian.kammerlander@med.uni-muenchen.de (C.K.); wolfgang.boecker@med.uni-muenchen.de (W.B.); carl.neuerburg@med.uni-muenchen.de (C.N.)

**Keywords:** pelvic fracture, fragility fractures, pelvic ring, pelvic ring fracture, insole-force sensors, weight-bearing, geriatric fracture

## Abstract

Background: Due to an aging society, more and more surgeons are confronted with fragility fractures of the pelvis (FFPs). The aim of treatment of such patients should be the quickest possible mobilization with full weight-bearing. Up to now however, there are no data on loading of the lower extremities in patients suffering FFPs. We hypothesized to find differences in loading of the lower limbs. Methods: 22 patients with a mean age of 84.1 years were included. During gait analysis with insole-force sensors, loading on the lower extremities was recorded during early mobilization after index fracture. Results: Especially the average peak force showed differences in loading, as the affected limb was loaded significantly less {59.78% (SD ± 16.15%) of the bodyweight vs. 73.22% (SD ± 14.84%) (*p* = <0.001, effect size *r* = 0.58)}. Furthermore, differences in loading in between the fracture patterns of FFPs were observed. Conclusion: This study shows that it is possible to reliably detect the extremity load, with the help of an insole device, in patients presenting with fragility fractures of the pelvis. There is great potential to improve the choice and time of treatment with insole-force sensors in FFPs in future.

## 1. Introduction

Fragility fractures of the pelvis (FFP) are one of the challenges in geriatric traumatology. The incidence of pelvic fragility fractures is steadily increasing, especially in the age group over 80 years [1]. In contrast to pelvic fractures of younger adults, which are usually caused by high-energy trauma such as severe road accidents, pelvic fragility fractures are caused by low-energy trauma, for instance a domestic fall. This can be primarily explained by reduced bone density and limited ligamentous stability in elderly patients [2]. There are two problems with the care of fragility fractures of the pelvis in older patients: first, the treatment of pelvic fractures is generally very demanding, even for experienced surgeons. This is made more difficult by reduced bone quality in the elderly. Secondly, older patients frequently present with various comorbidities, polypharmacy and muscle atrophy (sarcopenia), which aggravates frailty [3,4]. In these patients immobilization is a severe threat, as prolonged bedrest is associated with poor function at 2 months, and worsened survival at 6 months in elderly trauma patients [5]. Accordingly, the goal in diagnosing and treating these patients must be to make a decision as quickly as possible regarding surgery or conservative therapy, so that they can be mobilized and return to their daily activity with compensated pain status. This means that significant complications such as pneumonia, thrombosis and further muscle loss can be avoided during hospital stay and after discharge [6].

Differentiation of the fragility fractures of the pelvis can be made using the FFP classification by Rommens et al. [7,8,9]. Type FFP1 fractures are stable fractures that only affect the anterior pelvic ring. Type FFP2 are characterized by undisplaced fractures of the posterior pelvic ring, with type FFP3 showing displaced fractures on the posterior pelvic ring. In type FFP2 and FFP3, the anterior pelvic ring is usually also affected. Type FFP4 fractures are bilateral dislocated posterior pelvic ring fractures [8].

Diagnosing fragility fractures of the pelvis is difficult and in many cases the patients complain about pain, especially during movement [7]. Often there is no history of falls or trauma, or the patient cannot remember it due to dementia or cognitive impairment and the correct diagnosis might be delayed by this.

The pelvic CT scan is the work horse in diagnosing FFP, as it commonly shows the bony structures and cortical fracture lines which are often overseen in conventional radiographs [10]. Still MRI has great advantages as it can visualize bone marrow alterations such as edema or bone bruise. With 100% sensitivity for bone marrow alterations the MRI is proven to be the gold standard over conventional CT with 65–75% of sensitivity [11,12]. In daily clinical routine CT scans are usually ordered first and MRI is only performed when pain persists during mobilization.

A relatively new approach for diagnosing bone marrow edema in FFPs are dual-energy CT scans. Palm et al. describe in a recently published study that dual-energy CT scans are on par with MRI for sensitivity and specificity in diagnosing fragility fractures of the pelvis [13].

According to Rommens’ recommendation, FFP1 fractures should be treated primarily with a conservative approach. In principle, FFP2 fractures are also treated without surgery, while surgery is only recommended if there is no pain compensation and sufficient mobilization within one week. Surgical intervention is frequently recommended for type FFP3 and FFP4 fractures [8].

In cases in which it remains unclear which choice of treatment is best, and as an attempt to monitor mobilization outcomes in FFP patients, the use of new biosensor recorders to assess the ability of load-bearing can be beneficial. Thus, wearable insoles offer a novel and unique technology to gather metrics of gait in a potentially more clinically relevant way [14,15]. Wearable insoles have been used in different situations, such as aftercare of hip fracture patients [16,17] to measure weight-bearing and collect gait analysis data in elderly patients.

The purpose of this study was to evaluate the ability of a wearable insole sensor device to measure gait parameters in geriatric patients with FFPs. Further we sought to determine if the device was suitable and sensitive enough to identify potential subtle changes between the measures of different fracture patterns and treatment approaches.

## 2. Materials and Methods

### 2.1. Study Design and Participants

After receiving approval from the ethics committee (Ethikkommission bei der LMU München Ref.-No.: 19–292), a prospective observational trial was conducted to evaluate if it was possible to measure a difference between the fractured limb and the unaffected limb in patients that had suffered a fragility fracture of the pelvis. Patients seen for an FFP were considered for the study. Inclusion criteria were FFP I-IV, age > 70 years and a signed written consent. All subjects gave their informed consent for inclusion before they participated in the study. The study was conducted in accordance with the Declaration of Helsinki.

**Figure 1:**
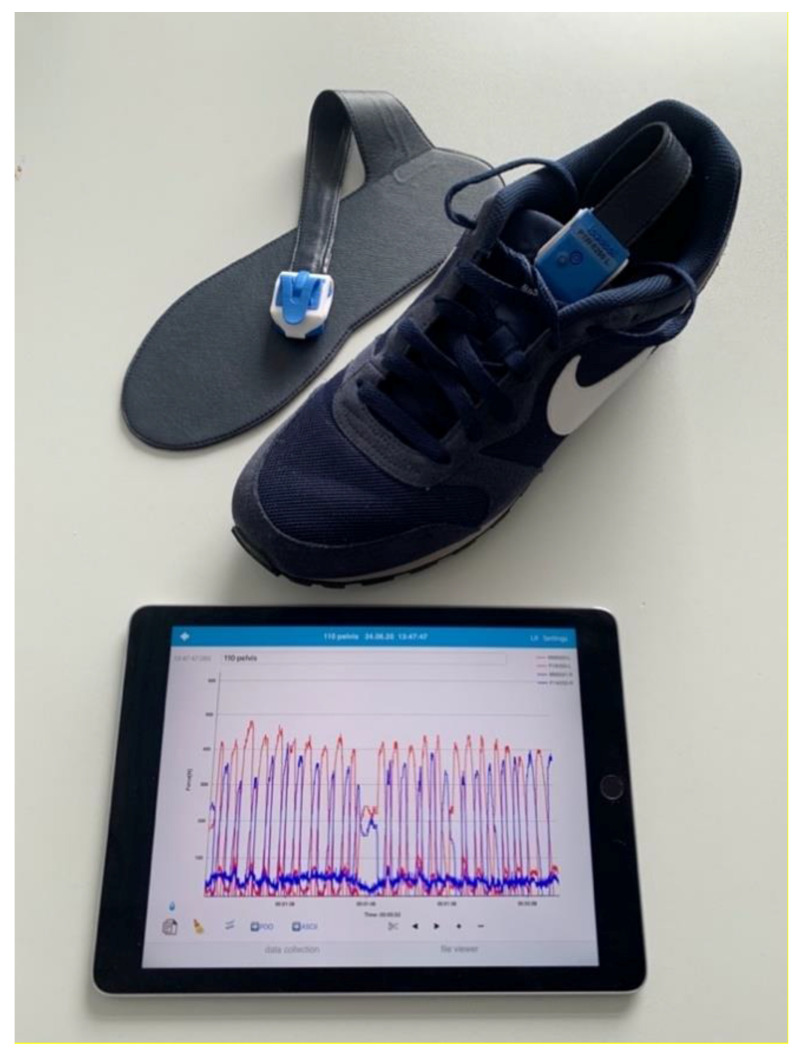
Setup of the Loadsol^®^ insole sensor device.

Those patients with a Minimal Mental State Exam (MMSE) lower than 27 were excluded in the present preliminary study approach in order to validate feasibility of the novel gait analysis approach for FFPs and to reduce differences in loading triggered by cognitive impairment and gait balance disorders. Immobility prior to the fracture or an additional fracture, even of the upper extremity, was an exclusion criterion. After the informed consent form was signed, the patients completed gait analysis using the Loadsol insole sensor device by Novel (Munich, Germany). Insole sensors were fitted into the shoe of each participant according to their appropriate shoe size (Figure 1). All sensors communicated via Bluetooth with an iPad (Apple, Inc., Cupertino, CA, USA). The decision to operate or not was made by three experienced consultants and the geriatrician in charge. For the operative patients, the measurement was done between 4–7 days post-operative. Standardized pain medication regimen according to WHO treatment guidelines was used for all patients. During gait analysis no local pain catheter was in use. Those patients who received conservative treatment for their fractures performed analysis 4–7 days after diagnosis. All patients were allowed the walking aid of their choice during measurement.

### 2.2. Insole-Force Sensors

Loadsol^®^ by Novel, was used in this study and has been shown to be a valid and reliable tool for wireless plantar force measurement in hopping, walking, and running [18]. Several studies were able to prove very similar results concerning the measurement with Loadsol^®^ compared to force plates [19,20].

**Figure 2:**
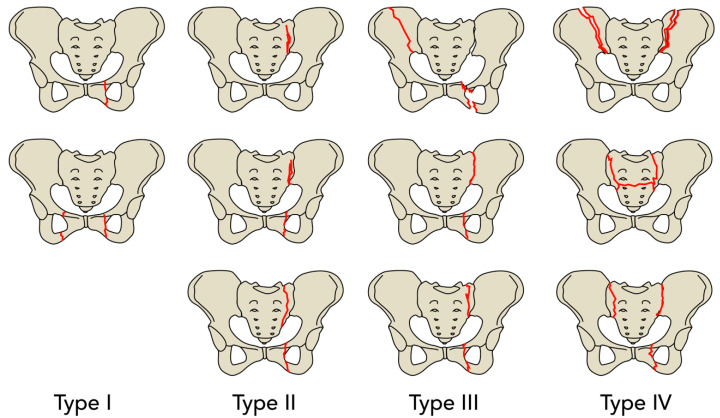
Comprehensive classification of fragility fractures of the pelvis according to Rommens and Hofmann [7]. Figure 1 is adapted from Ueda, Y. et al. [9] with permission from Springer Nature, 2020. FFP type I: anterior injury only. FFP type II: non-displaced posterior injury. FFP type III: displaced unilateral posterior injury. FFP type IV: displaced bilateral posterior injury.

**Figure 3:**
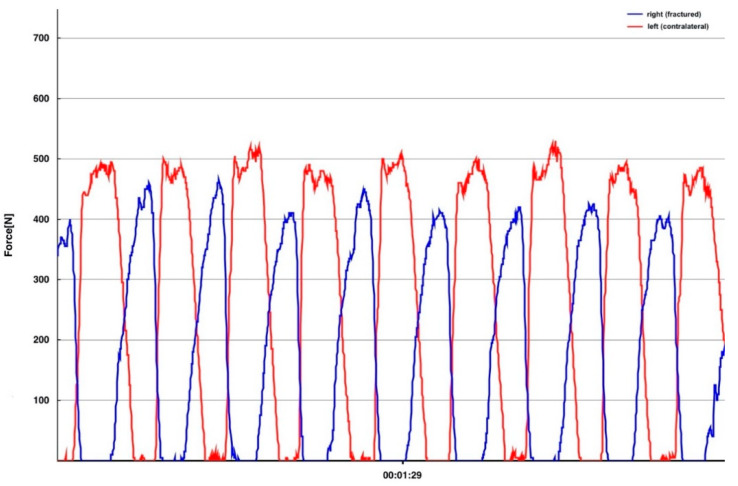
Displays the load in newton of the fractured side (blue graph) and the contralateral healthy side (red graph). Time in minutes is shown on the *x*-axis, while the *y*-axis represents force in newton. In this excerpt, 8 exemplary gait cycles are depicted.

The distance for the measurement was fixed at 40 m for all participants. Some of the FFP patients were not able to walk the whole distance due to persistent pain, in these patients the average loading was recorded on a walking distances as much as tolerated. The measurement included starting from a chair, level walking the distance, turning, and returning to the chair. Peak force, average loading rate and step count were measured and recorded for each foot separately (Figure 2). The insole devices are designed to cover the entire plantar surface of the foot and record the plantar force up to 200 Hz (Figure 3).

The max peak force is the main outcome in this gait analysis. Percent peak force is the percentage of the peak force of the affected side compared to the total body weight in Newtons. The percent load rate is measured as the percentage of the loading rate (N/s) of the affected side compared to the unaffected side. Percent load rate is measured as the percentage of the loading rate (N/s) of the affected side compared to the unaffected side. The loading rate of the feet was measured in Newtons per seconds. The loading rate is calculated as

(1) $$LR=\frac{\left(F80-F20\right)}{\left(t80-t20\right)}$$

where F20 and t20 are the Force in Newton (N) and time in second (s), measured when the force is at 20% of the heel impact peak. F80 and t80 are subsequently the force in Newton and time in seconds when the force is at 80% of the heel impact peak. An 11-point pain scale (0–10) was used to assess pain while walking and pain while resting. Additionally, the parker mobility score (PMS) and Barthel Index (BI) were collected from each patient.

### 2.3. Statistical Analysis

To check all data for normal distribution in advance of the analysis, the Shapiro–Wilk-Test was performed. Depending on the result of this test, either the Mann-Whitney-U-Test or the t-Test was used to identify significant differences between groups. When comparing FFP I and FFP IV patients the Mann-Whitney-U-Test was used for the percent avg. peak force and the *t*-Test was used for the percent max. peak force. Furthermore, the Wilcoxon rank sum test was performed to analyze the differences in the percent avg. peak force and the percent max. peak force comparing the fractured and the contralateral limb of each patient. To calculate the effect size of this test, the formula *r* = $\frac{Z}{\surd n}$ (Z = Z-Score, *n* = sample size) was used. The level of significance was set at *p* < 0.05. Patient characteristics were acquired using descriptive statistics.

Graphs and statistical analysis were calculated with IBM SPSS Statistics Version 25 (IBM Germany GmbH, Ehningen, Germany).

## 3. Results

Overall, 22 consecutive FFP patients were included within the trial with a mean age of 84.09 years (SD ± 5.98, range 73–95 years). 90.9% of the patients were female (20 female/2 male) with an overall mean weight of 58.66 kg (SD ± 8.04 kg), a mean BMI of 22.45 kg/m^2^ (SD ± 3.35 kg/m^2^) and a mean ASA Score of 2.68 (SD ± 0.65) (Table 1).

The fractures presented were classified according to the FFP classification with the following distribution: FFP I (*n* = 3, 13.6%), FFP II (*n* = 13, 59.1%), FFP III (*n* = 1, 4.5%) and FFP IV (*n* = 5, 22.7%). For an easier understanding of the results we pooled the FFP subtypes together in their main groups. Of all patients included, a total of *n* = 13 patients were treated conservatively (59.1%) over *n* = 9 patients with an operative treatment (40.9%) (Table 2).

The first set of analyses investigated whether we could find a difference in weight-bearing between the fractured side and the contralateral unaffected side. As seen in the percent Avg. Pf., the patients loaded the limb ipsilateral to the fractured side of the pelvis significantly less, with a mean of 59.78% (SD ± 16.15%) of the bodyweight in comparison to the contralateral limb with 73.22% (SD ± 14.84%) of the bodyweight (*p* = <0.001, effect size *r* = 0.58). A comparison of the percent Max. Pf. showed that the patients put a significantly lower maximum load on the affected limb with 75.10% (SD ± 13.64%) of the bodyweight, as opposed to the contralateral limb with 87.77% (SD ± 13.80%) of the bodyweight (*p* = <0.001, effect size *r* = 0.50) (Table 3).

A descriptive analysis of the gait parameters collected for each group of the FFP fracture types showed that the patients of each group loaded their affected side differently.

In view of the percent Avg. Pf. the patients with an FFP type I fracture loaded the affected side with 84.89% of the bodyweight (SD ± 23.14%), FFP type II patients reached a mean of 56.18% (SD ± 13.26%), 54.09% were measured for the FFP type III patient and 55.21% (SD ± 5.01%) in the FFP type IV group. Moreover, the percent Max. Pf. showed differences between the FFP groups examined (FFP I: 96.62% ± 21.99%, FFP II: 73.52% ± 8.80%, FFP III: 63.15% and FFP IV: 68.55% ± 7.59%). However, no such clear tendency can be seen in the percent Loading Rate (FFP I: 79.18% ± 63.94% vs. FFP II: 65.46% ± 23.24% vs. FFP III: 75.30% vs. FFP IV: 67.78% ± 19.43%), especially comparing FFP II and IV (Table 4).

Concerning ASA score, Barthel Index and Parker Mobility Score there was no deterioration detectable with regards to an increasing FFP type (Table 5).

Drawn from the descriptive analysis above, another promising finding was that the patients with a low grade FFP I fracture put a significantly higher load on their affected limb than the patients with a high grade FFP IV fracture. The percent Avg. Pf. in the FFP I group had a mean of 84.89% (SD ± 23.14%) in contrast to a mean percent Avg. Pf. of 55.21% (SD ± 5.01%) in the FFP IV group (*p* = 0.036). Furthermore, there was a significant difference in the percent Max. Pf. between both groups with a mean of 96.62% (SD ± 21.99%) in the FFP I group, versus a mean of 68.55% (SD ± 7.59%) in the FFP IV group (*p* = 0.035) (Table 6).

Beyond this finding, a comparison of the FFP I group, with fractures limited to the anterior pelvic ring, and the remaining groups FFP II-IV, with an obligate involvement of the posterior pelvic ring, resulted in a significant difference. Again, patients with a low grade FFP I fracture put a higher load on the affected limb than patients with a more severe FFP II-IV fracture. This was detectable in both the Avg. Pf. (FFP I: 84.89% ± 23.14% vs. FFP II-IV: 55.93% ± 11.11%, *p* = 0.002) and the Max. Pf. (FFP I: 96.62% ± 21.99% vs. FFP II-IV: 71.66% ± 8.58%, *p* = <0.001) (Table 7).

## 4. Discussion

The results of this study show that a wearable insole-force sensor was sensitive enough to detect differences in load-bearing between the affected and contralateral side, in patients that suffer a fragility fracture of the pelvis (FFP). In this analysis of FFP patients, the average peak force was the most sensitive gait parameter to detect differences in gait. This gait parameter is easy to understand, as it is the force between foot and shoe, which is measured in newtons and can be detected with different types of sensors [21]. The wearable insole device may prove to be a suitable technology to use in a clinical setting, even if this preliminary data only gives an idea of what this system is capable of.

The first important finding is that we detected the fractured side correctly in 100 percent of the patients. This is not as trivial as it sounds, considering that the clinical picture, radiological morphology and stability range widely [22]. A FFP, which is often difficult to detect correctly with a conventional X-ray [10] and therefore a more sensitive CT Scan is often recommended, can be detected correctly with an insole device. Although classification of FFPs and therapy recommendations have been proven, classification of FFP subtypes involving a complete non-displaced or displaced sacral fracture showed relatively poor inter-/intraobserver reliability which limits the usefulness of the FFP classification for both clinical and research purposes [23]. Thus, the present findings give an idea of how sensitive load-bearing detection and the clinical measurement of load can be used as a clinical tool.

It is likely that these fractures cause pain due to micro movements in the fracture zone, and therefore the patients try to reduce loading the fractured side. This goes along with the anatomical description of the FFP, where a higher FFP classification indicates a more unstable fracture type [24]. Referring to the fracture pattern and taking under consideration the degree of resulting instability, a conservative or operative treatment is generally recommended.

The second main finding is that the fractured side is loaded significantly less. The average Peakforce applied to the fractured side is significantly lower on the affected side than on the contralateral. Similar findings were described in previous studies using load measuring insoles to detect the plantar force in lower limb fractures [16,17,25]. To the best of our knowledge the present study is the first to investigate the use of a mobile insole sensor in fragility fractures of the pelvis.

A sub-analysis of FFP1 to FFP4 shows another promising finding; the patients with FFP I fractures put a significantly higher load on the fractured side than the patients with an FFP IV fracture. This sub-analysis gives an insight on how the load-bearing between different grades of pelvic instability are measurable with the insole sensor. Anterior versus posterior injuries also seem to have different load-bearing patterns. This is in line with the classification of Rommens and Hofmann, as they assume higher instability with higher fracture classification. In the present study patients suffering only anterior fractures load significantly more weight on the fractured side than patients suffering from combined anterior and posterior pelvic ring fractures.

The basic presumption is that early mobilization should be facilitated, and therefore a surgical intervention might be needed in higher degree FFPs. On the other hand, the recommendation for FFP2 is to be reevaluated within 5–7 days after the fracture was diagnosed and if the patient is still not mobile, surgery may be necessary. An insole sensor as a sensitive clinical tool might facilitate this process in future. Cut off values which would lead to surgical intervention or allow for conservative treatment cannot be determined in this comparatively small sample size. This will be of interest as the clinical pathway to find the right treatment for a patient suffering from an FFP is still under construction. Given the relative ease of measurement of parameters of gait, this could become a clinically usable instrument in future.

Early mobilization under pain treatment is the primary therapy goal in FFP 1 and 2, as geriatric trauma patients have a significantly increased mortality risk due to immobilization [5]. Thus, a multidisciplinary team of physiotherapists, geriatricians, nurses, and surgeons should conclude therapy decision in patients suffering FFPs. The early decision whether a conservative or surgical approach is needed, can be chosen through previous full mobilization and significantly reduces complications [26]. This should include physiotherapy treatment under pain-dependent full load [2].

The therapy decision is currently based on an individual assessment of the mobility of the patient. Physiotherapists and nurses who work closely with the patient can get very different impressions regarding mobility. Therefore, a wearable load measuring device such as the Loadsol, could be used to additionally measure the load applied to the fractured side.

This study is limited to early post-operative/post fracture time and the results cannot be extrapolated beyond the first week at this time. However, as the first week of early mobilization is the most important time frame, this is likely the best timing for the measurement, although a continuous measurement over the first 6 weeks should be the goal [5]. Although gait analysis with an insole-force sensor has proven its feasibility to provide additional insights in FFPs, the present study contains some limitations. Furthermore, data of the present study remains preliminary while the sample size is still small. Also patients with an MMSE < 27 were excluded from the present study although this remains a condition frequently observed in elderly patients suffering from FFPs we aimed to reduce loading differences triggered by cognitive impairment and gait balance disorders. Only one wearable insole design was evaluated, and while the sensor used in this study shows a high validity and is easy to use [18,19,20,27], there are other products on the market (e.g., Mediologic, Tekscan, Pedar) that could offer similar measurements, and may merit further study [21].

Future studies should also focus on how to find cut off values and how to differentiate between unstable and stable fractures and choice of surgical treatment by load measuring devices.

## 5. Conclusions

The present study primarily proved feasibility of insole-force sensors that provide new treatment insights in fragility fractures of the pelvis. Gait analysis detected the fractured side correctly in 100 percent of the patients, while the limb where the FFPs were located was loaded significantly less compared to the healthy contralateral side. Furthermore, patients having suffered a low grade FFP I put significantly higher load on the fractured side than patients presenting with an FFP IV fracture. Additionally, differences in loading were observed when comparing fractures affecting the anterior pelvic ring only, compared to combined anterior and posterior pelvic ring fractures. Although gait analysis with an insole-force sensor has proven its feasibility to provide additional treatment insights in FFPs, future studies should focus on how to find cut off values for conservative vs. surgical treatment and how to differentiate between the choice of surgical treatment.

## Figures and Tables

**Table 1:** Population Characteristics.

Characteristic	Mean ± SD
Age	84.09 ± 5.98
Female sex, *n* (%)	20 (90.9)
Weight (kg)	58.66 ± 8.04
BMI (kg/m^2^)	22.45 ± 3.35
ASA Score	2.68 ± 0.65

**Table 2:** FFP Classification and Treatment.

FFP Type	*n* (%)
FFP I	3 (13.6)
FFP II	13 (59.1)
FFP III	1 (4.5)
FFP IV	5 (22.7)
Treatment	
Surgery	13 (59.1)
Conservative	9 (40.9)

**Table 3:** Comparison fractured vs. contralateral limb: gait analysis.

Parameter	Limb	Mean ± SD	*p*-Value	*r*—Effect Size
Avg. Pf. (% of bodyweight)	Fractured	59.78 ± 16.15	<0.001	0.58
	Contralateral	73.22 ± 14.84		
Max. Pf. (% of bodyweight)	Fractured	75.10 ± 13.64	<0.001	0.50
	Contralateral	87.77 ± 13.80		

**Table 4:** Overall gait analysis.

Parameter	FFP I	FFP II	FFP III	FFP IV
Avg. Pf. (% of bodyweight)	84.89 ± 23.14	56.18 ± 12.26	54.09	55.21 ± 5.01
Max. Pf. (% of bodyweight)	96.62 ± 21.99	73.52 ± 8.80	63.15	68.55 ± 7.59
Percent Loading Rate (%)	79.18 ± 63.94	65.46 ± 23.24	75.30	67.78 ± 19.43

**Table 5:** Subgroup Characteristics.

Score	FFP I	FFP II	FFP III	FFP IV
ASA	2.67 ± 1.16	2.62 ± 1.64	3	2.80 ± 0.84
BI	65.00 ± 27.84	49.23 ± 13.20	75	67.00 ± 10.37
PMS	3.33 ± 1.16	2.23 ± 1.64	3	2.40 ± 1.14

**Table 6:** FFP I vs. FFP IV.

Parameter	FFP I	FFP IV	*p*-Value
Avg. Pf. (% of bodyweight)	84.89 ± 23.14	55.21 ± 5.01	0.036
Max. Pf. (% of bodyweight)	96.62 ± 21.99	68.55 ± 7.59	0.035

**Table 7:** FFP I vs. FFP II-IV.

Parameter	FFP I	FFP II-IV	*p*-Value
Avg. Pf. (% of bodyweight)	84.89 ± 23.14	55.93 ± 11.11	0.002
Max. Pf. (% of bodyweight)	96.62 ± 21.99	71.66 ± 8.58	<0.001
